# Topologically inferring pathway activity toward precise cancer classification via integrating genomic and metabolomic data: prostate cancer as a case

**DOI:** 10.1038/srep13192

**Published:** 2015-08-19

**Authors:** Wei Liu, Xuefeng Bai, Yuejuan Liu, Wei Wang, Junwei Han, Qiuyu Wang, Yanjun Xu, Chunlong Zhang, Shihua Zhang, Xuecang Li, Zhonggui Ren, Jian Zhang, Chunquan Li

**Affiliations:** 1 Department of Mathematics, Heilongjiang Institute of Technology, Harbin, 150050, China; 2 Department of Medical Informatics, Daqing Campus, Harbin Medical University, Daqing, 163319, China; 3 College of Bioinformatics Science and Technology, Harbin Medical University, Harbin, 150081, China; 4 Department of Biostatistics, Anhui Agricultural University, Hefei, 230030, China

## Abstract

Precise cancer classification is a central challenge in clinical cancer research such as diagnosis, prognosis and metastasis prediction. Most existing cancer classification methods based on gene or metabolite biomarkers were limited to single genomics or metabolomics, and lacked integration and utilization of multiple ‘omics’ data. The accuracy and robustness of these methods when applied to independent cohorts of patients must be improved. In this study, we propose a directed random walk-based method to evaluate the topological importance of each gene in a reconstructed gene–metabolite graph by integrating information from matched gene expression profiles and metabolomic profiles. The joint use of gene and metabolite information contributes to accurate evaluation of the topological importance of genes and reproducible pathway activities. We constructed classifiers using reproducible pathway activities for precise cancer classification and risk metabolic pathway identification. We applied the proposed method to the classification of prostate cancer. Within-dataset experiments and cross-dataset experiments on three independent datasets demonstrated that the proposed method achieved a more accurate and robust overall performance compared to several existing classification methods. The resulting risk pathways and topologically important differential genes and metabolites provide biologically informative models for prostate cancer prognosis and therapeutic strategies development.

The accurate prediction of disease status, responses to therapy, and clinical outcomes is a central challenge in clinical cancer research. The traditional diagnostic method, based on the analysis of morphologic characteristics of biopsy specimens, gives limited information and misses many important aspects of the tumor, such as its rate of proliferation and capacity for invasion and metastasis^1^. As high-throughput molecular profiling technologies evolve, molecular diagnostic methods are emerging to classify tumor samples and outperform traditional clinical parameters^2^. Advances in genomics and metabolomics have generated many candidate molecular biomarkers to improve the diagnosis and prognosis of pathologies^3^,^4^,^5^,^6^. However, the reproducibility of individual biomarkers in independent cohorts of patients has been questioned^7^,^8^,^9^. The prediction performance of some biomarkers identified in one dataset may be unsatisfactory for the same disease phenotype in an independent dataset. Various factors underlie this problem, including the cellular heterogeneity within tissues, the genetic heterogeneity across patients, the measurement error of platforms, and the noise in gene expression levels^10^,^11^,^12^, etc.

To address this problem, researchers have proposed pathway-based methods to identify robust pathway biomarkers at the level of functional categories^13^,^14^,^15^,^16^. For example, Guo *et al.*^13^ used the mean or median of the expression values of pathway member genes to infer pathway activity (the Mean and Median method), and then built classifiers using the pathway activities as the classification features. Lee *et al.*^14^ inferred pathway activity using condition-responsive genes (the PAC method). Condition-responsive genes (CORGs) are a subset of the pathway member genes whose combined expression values show optimal discriminative power between different disease phenotypes. This strategy improved the discriminative power of pathway activities by excluding the noise contributed by non-CORGs. Su *et al.*^15^ estimated pathway activity based on probabilistic inference. These pathway-based methods have shown better classification performance and demonstrated that high-level functional modules were more robust than individual gene markers. However, the pathway-based methods described above used the pathways as simple gene sets and ignored the topological information embedded in the pathway network. The genes in a pathway are unequal in their topological importance. The highly connected hub genes are often critical to the functionality of the whole pathway^17^. Their perturbation may have a greater impact on the pathway than that of less topologically important genes. However, the hub genes tend to show low but consistent changes in expression levels^18^. It has been reported that expression variability is an inherent property of a gene and the highly interacting hub genes display lower expression variability and expression noise^10^,^11^,^12^,^19^,^20^. The consistent differential signals of hub genes are often overshadowed by those of other non-hub genes whose changes may be unstable across samples, leading to less-robust pathway activities. Therefore, promoting the differential signals of hub genes according to their topological importance may contribute to a more robust pathway activity and better classification performance.

In our previous study, we proposed a directed random walk-based method (the DRW method) to evaluate the topological importance of genes, and weighted the genes at the pathway activity inference step^21^. This strategy improved the reproducibility power of the pathway activities and also the classification accuracy. Vaske *et al.* proposed the PARADIGM method, which also incorporated the pathway topology to infer patient-specific pathway activities from multi-dimensional cancer genomic data^22^. However, the existing methods are limited to single genomics. Because the initiation and progression of cancer is not only associated with the genetics of the patient but also other variables, such as an individual’s lifestyle and environment, classification methods based solely on genomics are far from comprehensive. Metabolomics studies the end results of the complex biological processes in a given cell, tissue, or organ. Therefore, metabolomic information can reflect the microenvironment of cells and their responses to drugs, environmental contaminants, and other factors. Integrative analysis of genomic and metabolomic data will thus help to comprehensively interpret the underlying biological phenomena. Recently, researchers have proposed several metabolic pathway identification methods, including Subpathway-GM^23^ and IMPaLA^24^ and have showed that integrating differential genes and metabolites allows a more accurate level of pathway analysis. However, few studies have focused on integrating genomic and metabolomic data for the inference of risk metabolic pathway activity and cancer classification.

In this study, we proposed a novel method to improve the accuracy of cancer classification by joint analysis of genomic and metabolomic data using directed random walk on a global gene–metabolite pathway graph (DRW-GM, Fig. 1). DRW-GM uses directed random walk to evaluate the topological importance of each gene in a reconstructed gene–metabolite graph by integrating information from matched gene expression profiles and metabolomic profiles. The topological importance of genes is used to weight the genes for the inference of robust DRW-GM-based pathway activities. We show that the DRW-GM-based pathway activities have greater reproducibility power than those inferred using only genomic information. DRW-GM mainly focuses on two-class classification problem, which is one of the most popular problems in biology, such as normal vs tumor. We apply the DRW-GM method to the classification of prostate cancer, including benign prostate samples (Benign) vs localized prostate cancer samples (PCA), and PCA vs metastatic prostate cancer samples (Mets). Both *within-dataset* experiments and *cross-dataset* experiments show that the DRW-GM method is more accurate and more robust than several existing pathway-based methods. Our classification method also identified many risk metabolite pathways and topologically important, differential genes that are biologically informative for the treatment of prostate cancer.

## Results

**Figure 1:**
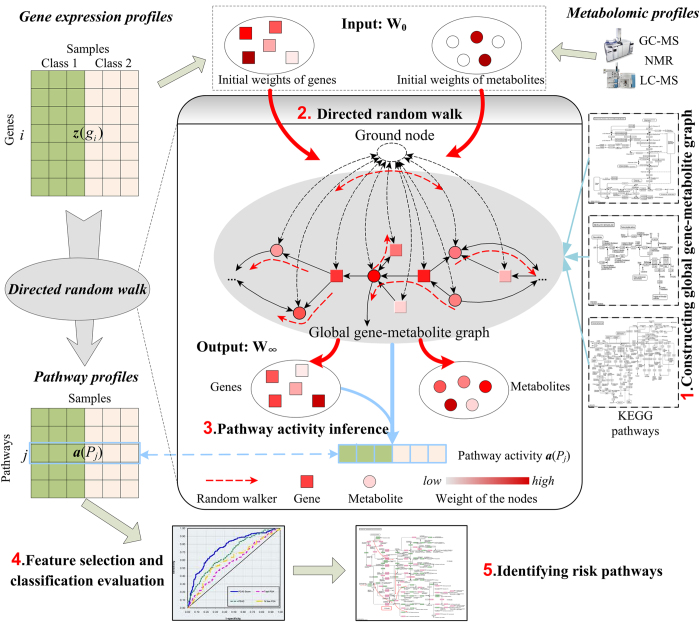
Schematic overview of DRW-GM. The gene expression profiles are translated into pathway profiles (rows represent pathways and columns represent samples) by DRW-based pathway activity inference. The global gene–metabolite graph is constructed on KEGG metabolite pathways. The input **W**_0_ of DRW is the initial weights of genes and metabolites, which are obtained from gene expression profiles and metabolomic profiles respectively. The output **W**_∞_ is the probability weight vector of nodes when DRW reaches a steady state. **W**_∞_ measures the topological importance of genes by incorporating both gene and metabolite information. The pathway activity is a expression value vector synthetized by topologically weighted differential gene expression vectors in the pathway (Formula (2)).

DRW-GM is a disease classification method which performs pathway-based classifier construction and precise disease status prediction by joint analysis of genomic and metabolomic data and pathway topology. Figure 1 shows a schematic overview of DRW-GM. DRW-GM evaluates the topological importance of genes by directed random walk on a global directed gene–metabolite pathway graph. The global directed gene–metabolite pathway graph is constructed based on the metabolic pathways in the Kyoto Encyclopedia of Genes and Genomes (KEGG, see Matierials and Methods). This global gene–metabolite graph includes 1353 genes, 1489 metabolites and 9824 edges. Using the topological importance of the genes, DRW-GM translates the gene expression profiles into pathway profiles with a pathway activity inference method (see Materials and Methods), and then selects the pathway activity features for classification validation and risk pathway identification.

We applied the DRW-GM method to the classification of prostate cancer, including Benign vs PCA and PCA vs Mets. The matched prostate metabolomic profiles^5^ and gene expression profiles (GSE8511) were integrated to evaluate the topological importance of the genes and to infer the pathway activities. We built classifiers based on the pathway activities and validated the classification performance using both *within-dataset* experiments and *cross-dataset* experiments. To confirm the effects of the joint analysis of genomic and metabolomic data, we compared DRW-GM with existing pathway activity inference methods that use only genomic information, including DRW^21^, PAC^14^, Mean and Median^13^. We also compared the classification performance with a traditional gene-based classifier that uses individual genes as features (the Genes method). Among these methods, DRW requires a gene-gene interaction network to evaluate the topological importance of genes, so to ensure a fair comparison, we also constructed gene-gene graphs based on the KEGG metabolite pathways (see Materials and Methods). The genes used to test the Genes method were the 1353 genes included in the global gene–metabolite graph.

### DRW-GM improves classification performance

To evaluate the utility of pathway activities in discriminating different disease phenotypes, we used pathway activities as the features with which to build the classifiers based on logistical regression model. We performed both *within-dataset* experiments and *cross-dataset* experiments to test the classification performance of the pathway activities.

#### Within-dataset experiment

We applied the DRW-GM method to the classification of the prostate cancer dataset GSE8511. This dataset contains samples of three phenotypes: Benign, PCA, and Mets. We used the classification to distinguish between Benign and PCA samples and between PCA and Mets samples. The area under the receiver operating characteristics curve (AUC) was used to measure the classification performance. We repeated five-fold cross-validations 100 times, and reported the mean of the resulting 1500 AUCs as the final AUC with which to evaluate the overall classification performance (see Materials and Methods). To compare the classification performance of different pathway activity inference methods, we performed the PAC, Mean, Median, and Genes method with the same *within-dataset* experimental set-up.

Table 1 showed a summary of the AUC of *within-dataset* experiments (Table S1 showed the classification accuracy). The DRW-GM method yielded the average AUCs (accuracies) of 0.9684 (90.12%) and 0.9992 (95.81%) in the “Benign–PCA” and “PCA–Mets” classification, respectively. Its overall classification performance was better than other five methods. Note that DRW-GM produced the smallest standard deviation (0.0834 and 0.0123, respectively), indicating the robustness of the DRW-GM-based pathway activities. The DRW-GM method also outperformed DRW in both the “Benign–PCA” and “PCA–Mets” cases. The difference between DRW-GM and DRW is that DRW-GM incorporates metabolomics information, suggesting that integrating genomic and metabolomics information increases classification accuracy.

#### Cross-dataset experiments

To evaluate the classification performance of the classifier on independent datasets, we further carried out *cross-dataset* experiments. The dataset GSE8511 was used as the training set, and the three datasets GSE3325, GSE32269, and GSE35988 were used as the test sets. All the datasets contained Benign, PCA, and Mets samples. As in the *within-dataset* experiment, we performed classification between the Benign and PCA samples, and between the PCA and Mets samples, respectively. To obtain an unbiased evaluation, we repeated the random partitions of the training sets 100 times to build the classifiers, and reported the mean of the resulting 500 AUCs as the final AUC that measured the classification performance on the independent test set (see Materials and Methods).

Table 2 showed the results of the *cross-dataset* experiments (Table S2 showed the classification accuracy). The DRW-GM method obtained AUCs (accuracies) of 0.9899 (93.60%), 0.8522 (81.96%), and 0.9836 (84.15%) in the “Benign–PCA” case and 0.9958 (91.60%), 0.9011 (60.89%), and 0.9994 (95.00%) in the “PCA–Mets” case on three independent test sets GSE3325, GSE32269, and GSE35988 respectively. The AUCs of the DRW-GM method ranked first in five of the six classifications of the paired training–test sets, indicating that the DRW-GM method yielded better overall classification performance. Although the Genes method obtained the best AUC (0.9023) on GSE32269 in the “Benign–PCA” case, its classification performance in the “PCA–Mets” case was not satisfactory. In the “PCA–Mets” case, the AUC of the Genes method (0.7492) on GSE32269 was much lower than that of the DRW-GM method (0.9011). The Genes method was also outperformed by the other pathway-based methods on the GSE3325 and GSE35988 datasets. This indicates that individual gene markers are less robust in predicting prostate cancer metastasis than pathway markers. This may be because the mechanism of prostate cancer metastasis is very heterogeneous. However, both the DRW-GM and DRW methods, which incorporate pathway topological information to infer pathway activities, yielded consistently superior performances in the “Benign–PCA” and “PCA–Mets” cases. The DRW-GM method again outperformed the DRW method because it incorporates metabolite information to evaluate the topological importance of genes. The metabolite information contributes in two ways: i) the use of metabolites to construct the global gene–metabolite graph; and ii) the use of differential metabolites to assist in evaluating the topological importance of genes at the process of random walking. To show this, we evaluated the classification performance of a modified DRW-GM method, referred to as DRW-GM-NM. DRW-GM-NM uses the gene–metabolite graph as the global pathway graph, but uses only the –log(p-value) of the genes (it does not incorporate the differential metabolites) as the initial weights to evaluate the topological importance of the genes. In the six experiments, the AUCs of DRW-GM-NM were larger than those of DRW, which uses a gene–gene graph, in five experiments (Table 2). This indicates that the gene–metabolite graph allows a more accurate estimation of topological importance than the gene–gene graph. We also found that DRW-GM was superior to DRW-GM-NM on almost all datasets (Table 2), confirming the contribution of differential metabolites to the process of random walking. In summary, the *cross-dataset* experiments described above show that the integration of genomic and metabolomic data improves the accuracy and robustness of the classification performance on independent datasets.

### DRW-GM provides biologically informative models for prostate cancer prognosis

We next analyzed the pathway activities that were selected to build the classifiers in the *cross-dataset* experiments described above (Table S3) and identified the frequently selected pathway markers (Table 3). We examined the differential genes that were used to infer the pathway markers (Table S4 for Benign vs PCA and Table S5 for PCA vs Mets) and sorted the differential genes by their topological importance (in descending order). The topological importance of the genes was measured with the topological weight vector **W**_∞_, which was calculated with DRW-GM on the global directed gene–metabolite graph (see Materials and Methods, Table S6). A larger weight indicates that a gene is more topologically important. The differential genes with topological importance rankings of <100 were listed for each pathway (Table 3; the full list was provided in Table S4). The gene *CYP1A1* (Entrez ID: 1543) in the steroid hormone biosynthesis pathway was the most frequently selected gene markers (1327/1500; Table S7) in the “PCA–Mets” case. *CYP1A1* plays an important role in the carcinogenesis of various cancers and many researchers have reported its association with the risk of prostate cancer^25^,^26^,^27^,^28^. We also sorted the differential genes by their p-values (*t*-test; Table S6), and compared the p-value rankings with the topological importance rankings (The topological rankings were not correlated with p-value rankings across all datasets we used; see Table S8~S10). The genes with elevated topological rankings also deserve attention. For example, purine nucleoside phosphorylase (*PNP*; Entrez ID: 4860) in the purine metabolism pathway had an increased ranking of 320 (topological ranking: 66; p-value ranking: 386; Table S5). The expression levels of *PNP* were upregulated in the Mets samples (p = 9.58 × 10^–3^). *PNP* is reported to be an oncogene and *PNP* knockdown experiments have shown that inhibiting *PNP* significantly reduces cell proliferation, migration, and invasion activity in prostate cancer cell lines^29^. Therefore, *PNP* inhibition was suggested as the target of a novel treatment for prostate cancer^29^. Another gene, spermine synthase (*SMS*; Entrez ID: 6611) in the arginine and proline metabolism pathway, also had an increased ranking of 320 (topological ranking: 64; p-value ranking: 384; Table S5). *SMS* is involved in the synthesis of polyamines, and patients with increased polyamine levels reportedly have poor prognoses^30^,^31^. Increased polyamine availability also enhances the capacity of cancer cells to invade and metastasize to new tissues^32^.

**Figure 2:**
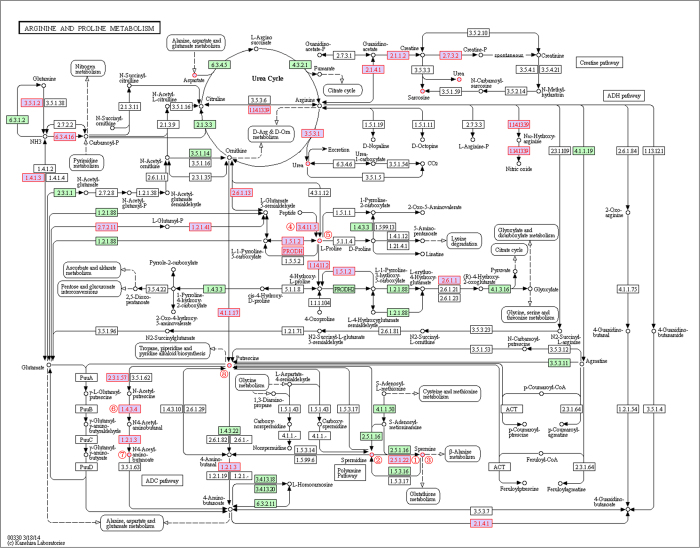
A snapshot of the perturbed nodes in arginine and proline metabolism pathway. The differential genes and metabolites of prostate cancer (PCA–Mets) are shown with red node labels and borders. The nodes discussed in Discussion section are marked with: ①: EC:2.5.1.22, SMS; ②: cpd: C00315, spermidine; ③: cpd: C00750, spermine; ④: EC:3.4.13.5, LAP3; ⑤: cpd:C00148, L-Proline; ⑥: EC:1.4.3.4, MAOB; ⑦: cpd:C02946, N4-Acetylaminobutanoate; ⑧ cpd:C00134, Putrescine.

**Figure 3:**
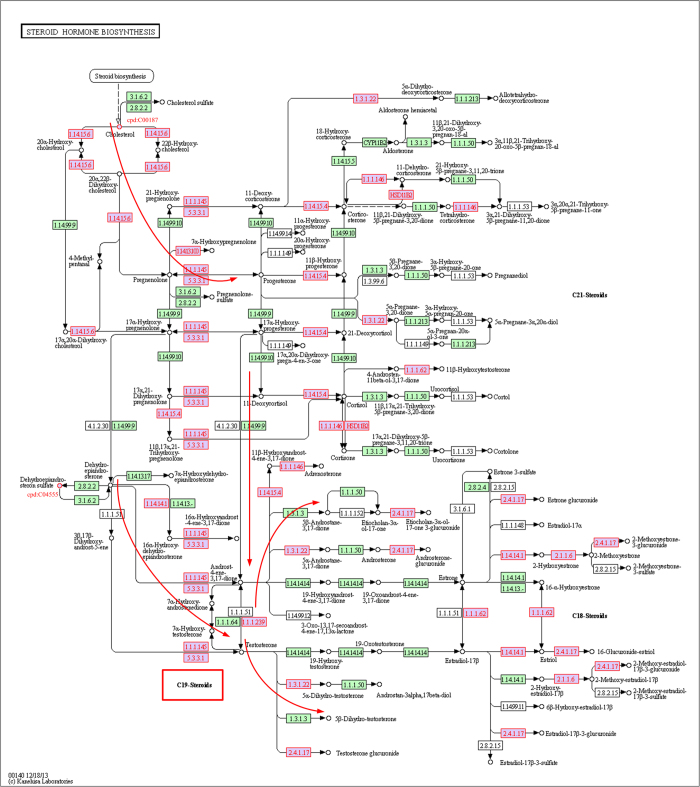
A snapshot of the perturbed nodes in steroid hormone biosynthesis pathway. The differential genes and metabolites of prostate cancer (PCA–Mets) are shown with red node labels and borders. The key enzymes in the pathway (red arrows) to form C-19 steroids of androgens are perturbed.

We also analyzed the differential metabolites in frequently selected risk pathways (Table 3). We sorted the metabolites by their topological importance (in descending order), which was measured with the topological weight vector **W**_∞_ (Table S11). Metabolites with high topological rankings may play important roles in prostate cancer. For example, l-Proline in the arginine and proline metabolism pathway ranked first in the “PCA–Mets” case (Tables S3 and S6). Combinations of proline and several other amino acids are reported to exert antitumor effects and become potent anticancer agents in the treatment of prostate cancer^33^. l-Proline is a differential hub node with many differentially expressed neighbors, including EC:3.4.11.5, EC:1.5.1.2, and EC:1.14.11.2 (Fig. 2, l-Proline is marked with ⑤). Therefore, it obtained the highest topological weight in random walking. These compact differential genes and metabolites constitute a dysregulated subpathway that may greatly affect the whole pathway. Cholesterol ranked third in the “PCA–Mets” case (Tables S3 and S6). It is located at the most upstream position in the steroid hormone biosynthesis pathway (Fig. 3) and is the starting reactant in the synthesis of androgen, the primary therapeutic target in the treatment of prostate cancer^34^,^35^. The recently identified prostate cancer biomarker sarcosine^5^,^36^,^37^ is involved in both the arginine and proline metabolism pathway and the glycine, serine and threonine metabolism pathway. It also has a high topological ranking (ranked 14th; Tables S3 and S6). We further found that the differential metabolites can assist in evaluating the topological importance of genes. For example, in the arginine and proline metabolism pathway, the gene *SMS* obtained a relatively large topological weight. Its topological ranking of 64 was much higher than its p-value ranking of 384 (see Results section, Table S5). *SMS* (EC:2.5.1.22, marked with ① in Fig. 2) is adjacent to two differential metabolites (cpd:C00315, spermidine and cpd:C00750, spermine, marked ② and ③ respectively) and participates in the polyamine synthesis pathway (Fig. 2), which is associated with prostate cancer metastasis^30^,^31^,^38^. The integration of the differential metabolites outstood the topological importance of *SMS*. Other topologically important differential genes in the arginine and proline metabolism pathway are also reinforced by differential metabolites. For example, *LAP3* (EC:3.4.13.5, topological ranking 8; Table S5, marked with ④ in Fig. 2) is close to l-Proline (cpd:C00148, marked with ⑤); and *MAOB* (EC:1.4.3.4; topological ranking 14, marked with ⑥) is close to N4-acetylaminobutanoate (cpd:C02946, marked with ⑦) and putrescine (cpd:C00134, marked with ⑧) (Fig. 2). The arginine and proline metabolism pathway was selected more frequently to build classifiers in DRW-GM (105/1500; Table S3) than in DRW (31/1500; Table S12). Another pathway, purine metabolism which contains five differential metabolites, was also selected more frequently in DRW-GM (222/1500; Table S3) than in DRW (39/1500; Table S12). The superior classification results for the DRW-GM-based pathway activities indicate that incorporating differential metabolites effectively improves the evaluation of topological importance and then the pathway activity.

We next investigated whether the identified risk pathways and their topologically important differential genes and metabolites can provide insight into the biological basis of prostate cancer. We focused on the steroid hormone biosynthesis pathway (ranked first), identified in the “PCA–Mets” case. It has been reported that changes in steroid metabolism contribute to the development of castration-resistant prostate cancer and underscore these pathways as critical targets of therapy^39^. As an androgen-dependent disease, the primary treatment of metastatic prostate cancer remains androgen deprivation therapy. However, castration does not eliminate androgens from the microenvironment of the prostate tumor^34^,^35^. Changes in the steroid enzymes may potentiate *de novo* androgen synthesis, promoting the progression of prostate cancer^40^,^41^. Therefore, we analyzed the process of androgen synthesis in the steroid hormone biosynthesis pathway in the PCA and Mets samples (Fig. 3). Steroid hormone synthesis begins with the transfer of a C-27 cholesterol molecule (cpd:C00187, located in the upper left corner of Fig. 3) from the outer mitochondrial membrane to the inner mitochondrial membrane. Importantly, the concentrations of cholesterol were significantly different (p = 3.05 × 10^–3^, Wilcox rank-sum test) in the metabolomic profiles of the PCA and Mets samples. Cholesterol is converted to C21 steroids by CYP11A (EC:1.14.15.6, p = 4.40 × 10^–2^). The C21 steroids are converted to the C19 adrenal androgens, dehydroepiandrosterone (DHEA) and androstenedione, by the sequential hydroxylase and lyase activities of CYP17A (EC:1.14.99.9). DHEA and androstenedione are then acted upon by 3 beta-hydroxysteroid dehydrogenase/Delta 5—>4-isomerase (EC:1.1.1.145/5.3.3.1; HSD3B1, p = 2.92 × 10^–2^) and Hydroxysteroid 17-beta dehydrogenase (EC:1.1.1.62/1.1.1.239; HSD17B1, p = 1.18 × 10^–3^; HSD17B6, p = 6.32 × 10^–8^, HSD17B2; p = 1.38 × 10^–2^), respectively, to form testosterone, which is converted to dihydrotestosterone by 3-oxo-5-alpha-steroid 4-dehydrogenase 2 (EC:1.3.1.22; SRD5A2, p = 2.53 × 10^–7^). We have marked the dysregulated enzyme nodes with a red node border (Fig. 3). Almost the entire enzymatic sequence in the pathway (red arrows in Fig. 3) that forms the C-19 androgen steroids are disturbed. To mediate androgen production, a multitargeting treatment that inhibits the critical components of this pathway, such as CYP17A (EC:1.14.99.9), SRD5A2 (p = 2.53 × 10^–7^), and HSD3B1 (p = 2.92 × 10^–2^), has emerged as a novel therapy, with impressive clinical outcomes^39^,^42^. This suggests that the risk pathways identified and their topologically important differential genes may provide novel hypothetical therapeutic targets for further biological experiments.

### DRW-GM improves the reproducibility power of pathway activities

**Figure 4:**
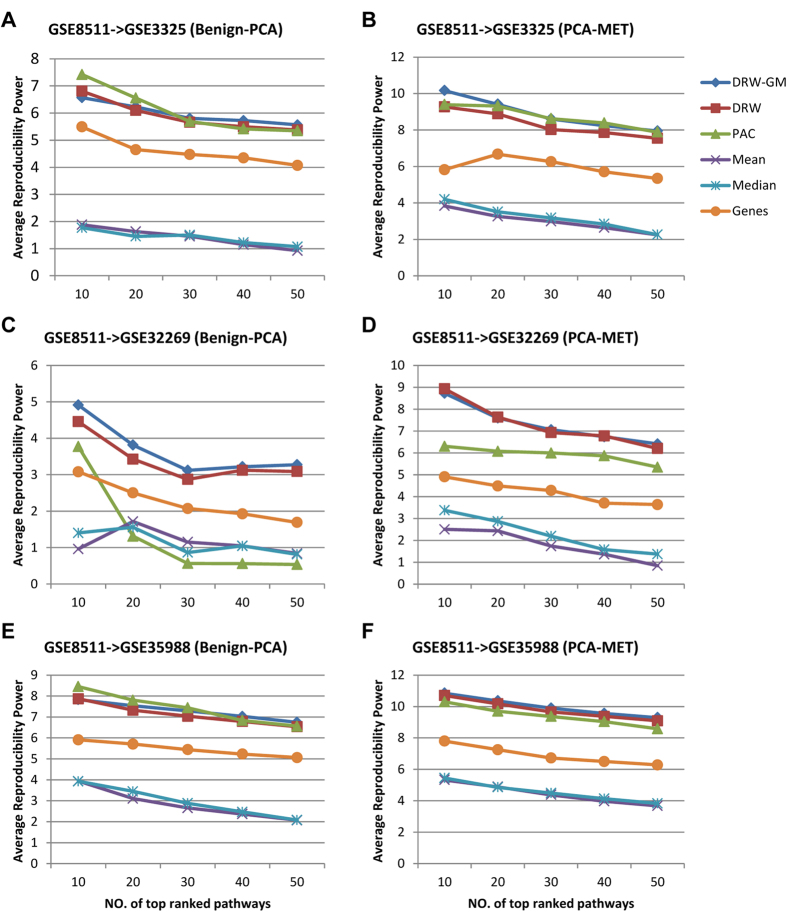
Reproducibility power of pathway activities. The reproducibility powers of pathway activities inferred by DRW-GM, DRW, PAC, Mean and Median were compared. The individual gene markers were also incorporated for comparison. (**A**,**C**,**E**) Comparison of reproducibility powers for Benign vs PCA (GSE8511->GSE3325, GSE8511->GSE32269, and GSE8511->GSE35988). The *x*-axis corresponds to the number *N* of top ranked pathways that considered, and the *y*-axis shows the reproducibility power *C*_*score*_ of the top *N* pathways. *N* = 10, 20, 30, 40, 50. (**B**,**D**,**F**) Comparison of reproducibility powers for PCA vs MET.

The generalization of a classifier and the practical use of biomarkers require that they have robust and discriminative features. We ranked the pathway activities by their absolute t-test score in descending order, and used the average reproducibility power of the top *N* pathway activities (*C*_*score*_(*N*), formula (4), see Materials and Methods) to evaluate the robustness of the pathway activities, where the reproducibility power of each pathway activity is defined as the product of its *t*-test score in the training set and that in the test set. *C*_*score*_ measures the consistency of the pathway activities between different data sets. A larger *C*_*score*_ value represents greater robustness and discriminative power. We tested the *C*_*score*_ values of the DRW-GM-based pathway activities between GSE8511 and three independent dataset (GSE3325, GSE32269, and GSE35988). To show that DRW-GM improves the reproducibility power of pathway activities, we also calculated the *C*_*score*_ values of the pathway activities inferred by the DRW, PAC, Mean, Median and Genes methods according to formula (4). We then compared the *C*_*score*_ values of the top *N* (*N* = 10, 20, 30, 40, or 50) pathway activities generated with the six methods. The *C*_*score*_ values of the DRW-GM-based pathway activities ranked first in 20 of 30 comparisons (numerical values are listed in Table S13). Wilcoxon signed-rank test showed that they were larger than those of the pathway activities inferred by the DRW, PAC, Mean, Median and Genes methods at the 5% significance level (Table 4). Although the top 10 PAC-based pathway activities showed larger *C*_*score*_ values in the GSE8511 → GSE3325 (Benign–PCA) (Fig. 4A) and GSE8511 → GSE35988 (Benign–PCA) (Fig. 4E) cases, the PAC-based pathway activities were not stable. Their *C*_*score*_ values were extremely low in the GSE8511 → GSE32269 (Benign–PCA) (Fig. 4C) and GSE8511 → GSE32269 (PCA–Mets) (Fig. 4D) cases. This may due to the strategy of PAC, which uses a greedy search to identify the most discriminative genes for pathway activity inference. The PAC method does not consider the topological importance of the genes. Therefore, it may incorporate some discriminative but unstable genes for pathway activity inference, which reduces the robustness of the pathway activity. The DRW-GM- and DRW-based pathway activities showed greatest robustness. Both methods incorporate topological information to weight the genes at the pathway activity inference step. The robustness of their pathway activities is enhanced by amplifying the differential signals of the topologically important genes. However, the *C*_*score*_ values of the DRW-GM-based pathway activities were significantly larger than those of the DRW-based pathway activities (p = 9.122 × 10^–6^, Table 4). Note that these two methods use the same genes to infer the pathway activities. The only difference between them is the strategy for establishing the topological weights used to weight the genes. DRW-GM incorporates information about differential metabolites into the evaluation of the topological weights of genes, whereas DRW uses only genomic data. This demonstrates that the integration of genomic and metabolomic information could help to evaluate topological weights of genes more accurately and infer more-robust pathway activities.

## Discussion

In this study, we have proposed a novel DRW-GM-based pathway activity inference method for the accurate and robust cancer classification via joint use of genomic and metabolomic information. The proposed method integrates genomic and metabolomic data to precisely evaluate the topological importance of genes in a reconstructed global gene–metabolite graph. The topological weights are used to weight the genes at the pathway activity inference step. Our results show that the DRW-GM-based pathway activities had higher reproducibility power and yielded superior classification performance. Topological weights play a critical role in pathway activity inference. The weighting strategy used to evaluate topological weights by integrating genomic and metabolomic data at the level of the pathway topology is the main difference between the DRW-GM and other pathway activity inference methods. For example, the Mean method^13^ assigns equal weights to all the genes in a pathway; the PAC method^14^ sets the weights of non-CORGs to 0, and assigns equal weights the CORGs. However, the importance of genes within the pathway is not equivalent. The changes in some key genes may greatly affect the pathway, whereas other genes that only appear somewhere downstream may do not affect the given pathway as much. In addition, some genes have stable expression changes in a disease. Their stable differential signals will contribute to the robustness of the pathway activities. However, these subtle differential signals can be easily overshadowed by other genes with larger but inconsistent variabilities across datasets if we assign them equal weights. Therefore, an adaptive weighting strategy that can amplify the stable differential signals of key genes is essential for inferring reproducible pathway activities.

DRW-GM improves the inference of pathway activities in three ways. First, it promotes the potential risk genes by integrating genomic and metabolomic information. Based on the assumption that genes associated with a similar disease or phenotype tend to be located in a specific neighborhood in the gene relationship network^43^,^44^, we use the t-scores of genes and differential metabolites to compute the initial weights of the nodes in the gene–metabolite graph. The initial weights are then redistributed according to the topological structure of the graph by directed random walk. The genes with large t-scores or near other differential genes and metabolites will receive more weights. The differential metabolites assist in evaluating the topological importance of the genes (*SMS*, *LAP3* and *MAOB* of arginine and proline metabolism pathway in Fig. 2). Second, DRW-GM promotes the hub genes. The hub genes are usually critical to the functionality of the whole pathway^17^. They tend to display small but stable expression changes. With their high connectivity, the hub genes will receive more weights at the process of random walk. Their stable differential signals are thus reinforced and contribute to the robustness of the pathway activity. Third, DRW-GM promotes upstream genes. Upstream genes are more topologically important as they could influence downstream genes^45^. They also have lower expression variability as expression noise can be transmitted from the upstream genes to downstream genes, adding substantially to the noise inherent in the expression of downstream genes^19^. Therefore, we reversed the direction of the edges in the gene–metabolite graph to make the weights flow to the upstream nodes. This strategy can help to evaluate the topological importance of genes more precisely and improve the classification performance^21^.

The initialization of weights of genes and metabolites can influence the final topological weights of genes, making it a key step of the directed random walk. For genes, we used the p-value from *t*-test to initialize the weights (See Materials and Methods). Other measures of relative expression can also be used, such as fold change. We tried to rerun the DRW-GM method using fold change instead of p-value, but did not get better classification performance on the same datasets (Table S14). For metabolites, considering that the metabolomic data is susceptible to environmental influence and the number of metabolites is relatively small, we used a strict cut-off (p < 0.01) to select differential metabolites and set their initial weights as 1 to enhance their effect at the process of directed random walk. We also performed the classification experiments using the p-value to initialize the weights of metabolites and obtained favourable classification performance. Nevertheless, it was slightly outperformed by our strategy that used a cut-off score to initialize the weights of metabolites (Table S14).

The incorporation of metabolites into the construction of the gene–metabolite graph also contribute to superior classification performance. In our comparison experiments, we performed the DRW-GM-NM method, in which the gene–metabolite graph is used for random walk. DRW-GM-NM yielded better classification performance than the DRW method, which uses a gene–gene graph (Table 2), indicating that the topological structure of the gene–metabolite graph is more accurate than that of the gene–gene graph. More complete biological pathway information clarifies the roles of the genes in the pathway and allows the topological importance of genes to be evaluated more accurately, improving the pathway activity and classification performance. The performance of DRW-GM-NM was inferior to that of DRW-GM (Table 2). Both methods use the same gene–metabolite graph for random walk. However, DRW-GM incorporats differential metabolites in the evaluation of topological importance, whereas DRW-GM-NM does not. This confirms the effect of differential metabolites on the process of random walk. Thus, the contribution of metabolomic data is two-fold. Metabolites are incorporated to construct the gene–metabolite graph and the differential metabolites incorporated in random walk are essential for improving the evaluation of topological importance, consequently improving the reproducibility power of the pathway activities and classification performance.

DRW-GM was implemented as an R package “DRWPClassGM”, available at http://222.170.78.208/DRW-GM/index.html and https://github.com/chunquanli/DRW-GM. It takes about 2 min to train a classifier on a personal computer with Inter i5 M430 CPU and 4G RAM. Although DRWPClassGM mainly focuses on two-class classification problems, it can be generalized to multiclass problem by decomposing the multiclass problem into multiple two-class problems. DRWPClassGM supports *t*-test and the significance analysis of microarrays method^46^ for differential genes identification and topological weights inference. We used *t*-test in our study considering that the majority of differential genes (two-tailed *t*-test, P < 0.05) did not show significantly different variances at the 1% significance level (two-tailed F test) in the four datasets (about 96.8%, 92.0%, 95.3% and 95.3% for the “Benign–PCA” case and about 85.9%, 89.1%, 80.6%, and 67.9% for the “PCA–Mets” case, in GSE3325, GSE32269, GSE35988 and GSE8511, respectively). DRWPClassGM also supports the construction of classifiers based on a user-defined pathway graph and new datasets. It is worth noting that, to ensure high prediction accuracy, DRW-GM requires enough differential genes in the new training set with which to build the classifier. If there is no genes with significant p-value or the number of differential genes number is less than a threshold (default is 50), the given dataset is considered as not discriminative and the algorithm will be stopped.

Although integrating genomic and metabolomic data has its advantage, the source of the metabolomic data must be carefully examined because metabolite concentrations are highly susceptible to environmental influence and extracted position of metabolomic data. Further, before integrating genomic and metabolomic data, one should ensure the robustness of metabolomic profiles, which is essential for accurately evaluating the topological importance of genes. For prostate cancer data in the paper, Sreekumar *et al.* assayed 42 tissue samples, 110 plasma and 110 matched urine samples of prostate cancer^5^. The result showed that the tissue metabolomic profiles exhibited more robust alterations. Therefore, for prostate cancer, we used their tissue metabolomic profiles in order to improve the stability of the result. Furthermore, as the functional biomarkers that identified at the level of functional categories or based on gene interaction network showed more robustness than the individual biomarkers^21^,^47^, our strategy of identifying pathway markers through integrating differential genes and metabolites based on the global gene-metabolite graph may alleviate the impact of the susceptibility of individual metabolites, contributing to more robust classification performance.

In addition, one thing to be aware of is that as most of the KEGG signalling pathways contain only a few detectable metabolites and the DRW-GM method need to use the differential metabolites for topological importance evaluation, we only use metabolic pathways for gene-metabolite graph construction and classification. This means that the DRW-GM method is limited in metabolic pathway analysis, and can not be used to identify risk signalling pathways.

Although metabolomic technology has improved, the global metabolomic profiling of prostate cancer is still in its infancy. The known metabolites account for only a small part of the molecules detected in metabolomic profiles. With the further development of metabolomic technology and the availability of more matched gene expression profiles and metabolomic profiles, we believe that integrating genomic and metabolomic information will allow a more accurate prediction of disease status and provide synergetic gene and metabolite biomarkers to guide clinical decision making.

## Materials and Methods

### Datasets

The unbiased metabolomic profiles, including 16 Benign, 12 PCA and 14 Mets, were obtained from the study of Sreekumar *et al.*^5^. These profiles detected more than 1126 metabolites using high throughput liquid and gas chromatography-based mass spectrometry (LC/GC–MS). We mapped these metabolite names to KEGG compound IDs and identified 145 known metabolites that also appeared in our KEGG metabolic pathway network. The matched gene expression profiles (GSE8511^48^: 16 Benign, 12 PCA, and 13 Mets) were downloaded from the Gene Expression Omnibus (GEO) database^49^. To evaluate the classification performance on independent datasets, we collected three more prostate gene expression datasets. Each dataset contained Benign, PCA and Mets prostate cancer samples (GSE3325^50^: six Benign, seven PCA, and seven Mets; GSE35988^51^: 16 Benign, 10 PCA, and eight Mets; and GSE32269^52^: four Benign, 22 PCA, and 29 Mets). The three gene expression datasets were detected with different microarray platforms. For each dataset, we normalized gene expression values to *z*-transformed scores, for which each gene *g*_*i*_ has a mean *μ* = 0 and s.d. *σ* = 1 over all the samples in the dataset. As the test sets, they not only validated the classification performance on independent datasets, but also across different platforms. Details of these datasets are provided in Supplementary Table S15.

## Methods

The workflow of DRW-GM is illustrated in Fig. 1: (i) construction of the global directed gene–metabolite graph; (ii) evaluation of the topological importance of the genes by directed random walk; (iii) inference of the pathway activity; (iv) feature selection and classification evaluation; and (v) identification of the risk metabolite pathways. The method is described in detail as follows.

### Constructing the global directed gene–metabolite graph

The gene–metabolite graph was constructed based on biochemical interaction information available in KEGG. First, all 150 metabolic pathways in KEGG were converted into directed graphs. For each pathway, we extracted all the enzymes and metabolites within the pathway as nodes. The edges were constructed from reactions. If a metabolite participated in a reaction, we connected the metabolite node and the enzyme node (i.e., reaction node) by a directed edge. The edge was directed from the metabolite node to the enzyme node if the metabolite acted as a substrate, and from the enzyme node to metabolite node if the metabolite acted as a product. For reversible reactions, we constructed two directed edges with opposite directions between the metabolite node and the enzyme node. Second, the enzyme-metabolite graph was expanded to a gene–metabolite graph. An enzyme node can map to multiple genes. We expanded the enzyme nodes to the corresponding gene nodes and each corresponding gene node was assigned the same connectivity and topological location as the corresponding enzyme node. Third, the total 150 graphs were merged into a global directed pathway graph, in which each node only contained one gene (metabolite) and each gene (metabolite) only appeared once. To perform DRW and highlight the importance of the upstream genes (metabolites), we reversed the direction of all the edges on the merged gene–metabolite graph and introduced a ground node connected to every node through bidirectional edges. The final gene–metabolite graph was denoted *G*.

For comparison with the DRW method^21^, which uses only gene expression information, we also constructed a global directed gene–gene graph of metabolic pathways based on the SubpathwayMiner software package^21^,^53^. The construction method was described in our previous study^21^.

### DRW-GM on the global directed gene–metabolite graph

The DRW method runs on a directed graph^21^. It simulates a random walker that starts on a source node *s* (or a set of nodes simultaneously), and transitions from its current node to a randomly selected neighbor or goes back to source node *s* with a restart probability *r* at each time step. The DRW method is defined formally as:


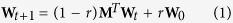


where **M** is the row-normalized adjacency matrix of the graph *G*, **W**_*t*_ is the weight vector in which the *i*-th element represents the probability of being at node *i* at time step *t*, and **W**_0_ is the initial weight vector of the nodes in *G*. Because there are two types of nodes in the gene–metabolite graph, the initial weights of the genes and metabolites must be assigned individually (the initial weight of the ground node is set to 0). We constructed **W**_0_ as follows:

(1) Constructed the weight vector of genes **WG**, in which the *i*-th element represented the weight of gene *g*_*i*_. The weight of gene *g*_*i*_ was set to –log (*p*_*gi*_), where *p*_*gi*_ was the p-value from a two-tailed *t*-test on its expression values between two phenotypes;

(2) Calculated the normalized weight vector of genes **WG′**. We rescaled the elements of **WG** between 0 and 1 with **WG′** **=** (**WG **− min(**WG**))/(max(**WG**) − min(**WG**)), where **WG′** was the normalized weight vector of the genes;

(3) Constructed the weight vector of metabolites **WM**, in which the *i*-th element represented the weight of metabolite *m*_*i*_. We incorporated the information of differential metabolites to improve the assessment of the topological importance of the genes. The differential metabolites were obtained from the metabolomic profiles of Sreekumar *et al.*^5^ using the Wilcoxon rank-sum test (p < 0.01; Table S16). We set the weight of each differential metabolite (p < 0.01) to 1, and the weights of other metabolites to 0. Therefore, genes closer to more differential metabolites obtained higher weights at the steady state of random walk.

(4) Constructed the initial weight vector **W**_0_. We combined **WG** and **WM** as 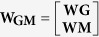 and normalized **W**_**GM**_ to a unit vector with 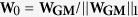, where 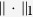 is the *L*_1_ norm.

At each time step, **W**_*t*_ is updated according to the formula (1). It proves that **W**_*t*_ will converge to a steady state **W**_∞_ after certain steps in the presence of the ground node^54^. We obtained the steady state **W**_∞_ by performing the iteration until the change between **W**_*t*_ and **W**_*t*+1_ (measured by the *L*_1_ norm) fell below 10^−10^. **W**_∞_ gave a measure of the topological importance of the genes in the global metabolic pathway graph *G*. The genes that are significantly differentially expressed and close to many differential genes and metabolites will obtain higher weights. These weights are used to weight the genes at the step of pathway activity inference. The stable differential signals of topologically important genes will be enhanced and contribute to the inference of robust pathway activities (classification features).

### Pathway activity inference

For each metabolic pathway, we selected the differential genes (p < 0.05; *t*-test) in the pathway to infer the pathway activity. For a pathway *P*_*j*_ that contains *n*_*j*_ differential genes {*g*_1_, *g*_2_, …, *g*_*nj*_}, the pathway activity ***a***_*T*_(*P*_*j*_) in the training dataset is defined as:


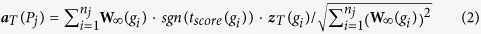


where **W**_∞_(*g*_*i*_) is the weight of gene *g*_*i*_ from random walk, sgn() is the sign function, *t*_*score*_(*g*_*i*_) is the *t* statistics of *g*_*i*_ from a two-tailed *t*-test on its expression values between two phenotypes, and ***z***_*T*_(*g*_*i*_) is the normalized expression value vector of *g*_*i*_ across the training samples. The pathway activity ***a***_*V*_(*P*_*j*_) in the test dataset is calculated by:


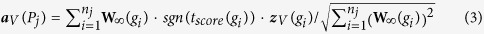


where ***z***_*V*_(*g*_*i*_) is the normalized expression value vector of gene *g*_*i*_ across the test samples.

For each sample, pathway activity is a derived score using the expression values of genes in the given pathway. Pathway activity ***a***(*P*_*j*_) is a synthetic expression value vector of *P*_*j*_ across the samples. Pathway activity inference transforms the gene expression profiles into pathway activity profiles (Fig. 1), which are then used to train a classifier.

### Reproducibility power

The reproducibility power^55^ measures the consistency of discriminant power between the pathway activities in the training set and the pathway activities in the independent test set. We ranked the pathway activities by their absolute *t*-test score (*t* statistic from a two-tailed *t*-test of pathway activity between two phenotypes) in the training set (in descending order). The reproducibility power is then defined as:


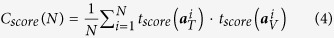


where *N* is the number of top-ranked pathway activities, *t*_*score*_(***a***) is the *t*-test score of pathway activity ***a***, 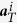 is the *i*-th pathway activity in the ranked pathway activities in the training set, and 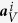 is the corresponding pathway activity in the test set. *C*_*score*_(*N*) reflects the averaged robustness and discriminative power of the top *N* pathway activities.

### Feature selection and classification evaluation

We performed a greedy search to select the pathway activity features and then constructed classifiers based on logistic regression model. For *within-dataset* experiments, we used five-fold cross-validation to evaluate classification performance. The dataset was randomly split into five equal-sized subsets. Each subset was used in turn as the test set, and the remaining four subsets were used as the training set. To select the best pathway features for classification, we further split the training set so that two thirds of samples in the training set were used as the *feature evaluation set* to rank the pathway features and train the classifier, whereas the remaining one third samples were used as the *feature selection set* for assessing which pathway feature set yielded the best classification performance. The pathway features were ranked by their *p*-values (*t*-test of pathway activities across samples in the *feature evaluation set*) and added sequentially to build the classifier. The performance (AUC) of the classifier was measured on the *feature selection set*. The added pathway feature was retained in the feature set if the corresponding AUC increased and was removed otherwise. The final feature set that produced the best AUC on the *feature selection set* was used to build the optimized classifier and to evaluate classification performance on the test set. For each training set, we built three optimized classifiers and obtained three AUCs on the corresponding test set. Thus, we generated 15 AUCs at the whole process. For an unbiased evaluation, we repeated the process described above 100 times. The mean of the resulting 1500 AUCs was reported as the final AUC with which to evaluate the overall performance of the classification method.

To evaluate the classification performance of the pathway activities on independent datasets, we performed *cross-dataset* experiments. Unlike some existing methods^14^,^15^ that use one dataset to select features and the other dataset to build the classifier and evaluate its performance, we used the whole first dataset as the training set and the second independent dataset as the test set. Our strategy is more realistic because we often do not have known homogeneous cohorts for a new patient in practice. As in the *within-dataset* experiments, the training dataset was randomly divided into five subsets of equal size. Each subset was used in turn as the *feature selection set*, and the remaining four subsets were used as the *feature evaluation set*. The optimized classifier constructed from the *feature evaluation set* was evaluated on the test set. One partition generated five AUCs. We repeated the random partitioning of the training set 100 times, and reported the mean of the resulting 500 AUCs as the final AUC with which to evaluate the classification performance on the independent dataset.

## Additional Information

**How to cite this article**: Liu, W. *et al.* Topologically inferring pathway activity toward precise cancer classification via integrating genomic and metabolomic data: prostate cancer as a case. *Sci. Rep.* **5**, 13192; doi: 10.1038/srep13192 (2015).

## Supplementary Material

Supplementary Information

## Figures and Tables

**Table 1:** Comparison of classification performance within datasets.

**Methods**	**Benign–PCA**	**PCA–Mets**
DRW-GM	**0.9684** ± 0.0834	**0.9992** ± 0.0123
DRW	0.9581 ± 0.1037	0.9980 ± 0.0281
PAC	0.9078 ± 0.1520	0.9473 ± 0.1165
Mean	0.8389 ± 0.1845	0.8275 ± 0.2083
Median	0.8224 ± 0.1859	0.8581 ± 0.1854
Genes	0.8436 ± 0.2007	0.8654 ± 0.1997

Shown are the average AUC and the standard deviation. The classification evaluation is performed according to *within-dataset* experiments on GSE8511. The AUC shown in bold is the best AUC for the corresponding two phenotypes (Benign–PCA and PCA–Mets).

**Table 2:** Comparison of classification performance cross datasets.

**Training set**	**Test set**					
**GSE8511**	**Benign–PCA**			**PCA–Mets**		
	**GSE3325**	**GSE32269**	**GSE35988**	**GSE3325**	**GSE32269**	**GSE35988**
DRW-GM	**0.9899** ± 0.0294	0.8522 ± 0.1990	**0.9836** ± 0.0216	**0.9958** ± 0.0170	**0.9011** ± 0.0724	**0.9994** ± 0.0070
DRW-GM-NM^a^	0.9866 ± 0.0363	0.8413 ± 0.2080	0.9817 ± 0.0271	0.9947 ± 0.0187	0.8995 ± 0.0786	0.9991 ± 0.0073
DRW	0.9780 ± 0.0404	0.8254 ± 0.2113	0.9827 ± 0.0196	0.9851 ± 0.0419	0.8918 ± 0.0750	0.9989 ± 0.0081
PAC	0.9634 ± 0.0643	0.8139 ± 0.2093	0.9675 ± 0.0405	0.9482 ± 0.1035	0.6645 ± 0.0836	0.9911 ± 0.0300
Mean	0.9450 ± 0.0733	0.6663 ± 0.2530	0.9351 ± 0.0446	0.9105 ± 0.0298	0.5172 ± 0.0788	0.9713 ± 0.0274
Median	0.9402 ± 0.0838	0.5315 ± 0.2121	0.8995 ± 0.0793	0.9036 ± 0.0671	0.7295 ± 0.0689	0.9694 ± 0.0512
Genes	0.9014 ± 0.1231	**0.9023** ± 0.1321	0.9682 ± 0.0186	0.8609 ± 0.0703	0.7492 ± 0.0835	0.8654 ± 0.0744

Shown are the average AUC and the standard deviation. The classification evaluation are performed according to *cross-dataset* experiments. The training set is GSE8511. Three independent test sets are GSE3325, GSE32269 and GSE35988. The classifications between Benign and PCA samples, and between PCA and Mets samples are carried out respectively. The AUC shown in bold is the best AUC for the corresponding paired training-test dataset.

^a^DRW-GM-NM: The classification method that uses gene–metabolite graph, but not incorporates differential metabolites for topological importance evaluation.

**Table 3:** Frequently selected pathway markers for prostate cancer prognosis.

**Pathway name**	**Frequency**	**Topologically important differential genes and metabolites**
Benign–PCA		
Porphyrin and chlorophyll metabolism	1030/1500	GUSB, FTH1, HMBS, ALAD, *Glycine*^a^
Purine metabolism	416/1500	POLR2H, PAICS, POLR3GL, NUDT9, NME1, AK2, NT5C, NME2, PAPSS1, POLR1A, PDE4A, GUCY1A3, ENTPD5, ENTPD3, PGM1, ADCY2, NUDT5, ITPA, POLR1E, *Glycine*
Pentose and glucuronate interconversions	62/1500	ALDH2, GUSB, AKR1B1
Drug metabolism - other enzymes	49/1500	UCK2, GUSB, TK1, ITPA
One carbon pool by folate	40/1500	AMT, MTHFD2, SHMT2
PCA–Mets		
Steroid hormone biosynthesis	701/1500	HSD17B6, SRD5A2, CYP1A1, *Cholesterol*, *Dehydroepiandrosterone sulfate*
Purine metabolism	222/1500	NT5C2, PDE8B, POLD1, NME5, PGM1, PNP, ADK, ADSL, POLR2J2, PKM2, NT5M, NT5C1A, ADCY2, ALLC, PDE6A, *Adenosine*, *Inosine*, *Urea*, *Urate*, *Guanosine*
Tryptophan metabolism	131/1500	MAOB, ALDH7A1, ACAT1, ALDH3A2, ALDH1B1, CCBL1, CYP1A1, ALDH2, *Tryptophan*
Arginine and proline metabolism	105/1500	LAP3, MAOB, ALDH7A1, ARG2, ALDH3A2, ODC1, ALDH1B1, SMS, GOT2, ALDH2, PYCRL, *Putrescine*, *L-Proline*, *L-Aspartate*, *Sarcosine*, *Urea*, *Spermidine*, *Spermine*, *4-Acetamidobutanoate*
Arachidonic acid metabolism	89/1500	PLA2G4A, HPGDS, PLA2G2D
Histidine metabolism	68/1500	MAOB, ALDH7A1, ALDH3A2, ALDH1B1, HNMT, HDC, ALDH2, *L-Aspartate*
Glycine, serine and threonine metabolism	53/1500	AOC3, MAOB, ALDH7A1, SHMT2, SRR, DLD, *L-Aspartate*, *Sarcosine*, *O-Phospho-L-serine*, *L-Serine*, *Tryptophan*
Drug metabolism - other enzymes	45/1500	DPYD, TK1, TYMP

^a^Metabolites are shown in italics.

**Table 4:** Comparison of reproducibility powers of six pathway activity inference methods.

	**DRW-GM vs DRW**	**DRW-GM vs PAC**	**DRW-GM vs Mean**	**DRW-GM vs Median**	**DRW-GM vs Genes**
p-value^a^	9.122 × 10^–6^	2.301 × 10^–4^	9.313 × 10^–10^	9.313 × 10^–10^	9.313 × 10^–10^

^a^Wilcoxon signed-rank test on the 30 Cscore values of DRW-GM against those of the other five methods.
